# The Hospital. Nursing Section

**Published:** 1906-10-06

**Authors:** 


### The Hospital.

**Nursing Section.**

Contributions for "THE HOSPITAL," should be addressed to the EDITOR, "THE HOSPITAL" NURSING SECTION, 28 & 29 Southampton Street, Strand, London, W.C.

No. 1,046.—VOL. XLI SATURDAY, OCTOBER 6, 1906.

### Notes on News from the Nursing World.

### OUR CLOTHING DISTRIBUTION.

A PROMPT and gratifying response has followed our appeal for contributions to enable us to make our Clothing Distribution at Christmas, for the benefit of poor patients in hospitals and infirmaries, even larger than it has been on previous occasions. Parcels have reached us from Nurse Mary Besant, London, and from No. 4219 Royal National Pension Fund. This is an early beginning, and will, we hope, act as a stimulant upon other readers to send in their gifts as soon as possible. All parcels must be addressed to the Editor, 28 and 29 Southampton Street, Strand, London, W.C., with "Clothing Distribution" marked on the outside.

### MISS NIGHTINGALE AND NURSING IN GERMANY.

THE GRAND DUCHESS OF BADEN, speaking at the opening of the International Congress for Cancer Research at Heidelburg, alluded to the work of Miss Florence Nightingale, and said that the British system of nursing, of which Miss Nightingale was the pioneer, has been borrowed by German institutions, to the great advantage of the latter. It is pleasant to know that Miss Nightingale's labours are not forgotten in Germany, and that the German people appreciate the English standard and methods of nursing so far as to adopt and make use of them—at any rate, to some extent.

### PRESENTATION TO MISS HULME.

ON Saturday last, the day of Miss Hulme's departure from the Nurses' Hostel, she was presented by a large number of nurses, representing both the north and south blocks, with a beautiful photogravure, framed handsomely in dark oak, of Raphael's masterpiece, "The Transfiguration." A small brass plate at the foot of the picture bore the words "Presented to Miss Hulme by the nurses of the Hostel as a small tribute of affection and esteem." This presentation was made in the sitting-room of the north block, where the nurses had assembled, and Miss Hulme, with obvious emotion, thanked them for their gift, which, she said, she should greatly value. This presentation affords another proof that Miss Hulme, in the short period she was at the Hostel, won the hearts of the nurses and made herself very popular, a fact of which additional evidence is supplied in our correspondence columns to-day. As the result of further inquiries respecting the circumstances of Miss Hulme's dismissal from the post of superintendent, we learn that, though her tenure of office did not expire until Saturday, the 29th, Miss Wood, the managing director, returned to the Hostel on Saturday, September 22, deprived Miss Hulme of the keys of the safe, degraded her from her position at the head of the table, and refused to allow her to take any share in the work of the office, or to officiate in chapel by reading prayers or playing the harmonium. Miss Hulme, we understand, was informed that Miss Wood acted in this manner by direction of the chairman, because of a letter which he had received from the nurses—many of them shareholders—asking that Miss Hulme might have three months' trial. It appears, too, that on Monday morning a notice was put up in the Hostel to the effect that the nurses were invited to meet Miss Wood in the sitting-room in the north block at 3 P.M. that day. Though the notice was exceedingly short, between 20 and 30 nurses were present, and they expected to hear from Miss Wood a statement of the cause of the dismissal of the late superintendent. The only definite statement forthcoming from Miss Wood was, however, that Miss Hulme had "a tendency to make the Hostel into a Nurses' Institution." But as a Board meeting has since been called for Friday this week, and it has been intimated that a deputation of nurses will be permitted to attend, we conclude that the official explanation has merely been deferred.

### A CHARGE OF SECTARIANISM.

WE publish a letter from a correspondent under the heading of "Sectarianism in London Hospitals" because the writer sends us, in confidence, the name of the school in which she was trained. We think, however, that whatever may have been the experience of "Alpha," the present matron of the institution to which we refer would not decline to promote the senior staff nurse to the post of sister because she happened to be a Romanist. Though it is open to institutions to refuse to receive Roman Catholics as probationers or to employ them as sisters, we should consider the exclusion both narrow-minded and impolitic. But to accept them as probationers, make them permanent staff nurses, and refuse to promote them to be sisters would be flagrantly unjust. We cannot, without the most convincing evidence to the contrary, believe that such a course would be pursued in any great London hospital or Poor-law infirmary at the present time.

### A DEMAND FOR STATE MIDWIVES.

IT will be seen from our report of the Conference of Midwives at Dusseldorf that the German midwives are not backward in urging their claims to recognition. A petition is to be presented to the authorities asking that they may be made public officials paid by the State. The answer to the demand will be awaited with interest in this country, where there is already a danger that the dubious progress made under the Midwives Act may lead to a similar cry.

### INCREASE OF STAFF AT WORCESTER INFIRMARY.

At a meeting of the Executive Committee at Worcester General Infirmary last month the question of augmenting the number of nurses was fully considered. The chairman stated that the request for the addition came from the surgical staff, who complained that the arrangements which had hitherto prevailed in the theatre were not satisfactory, owing to the increasing number of cases. The theatre had been under the charge of a theatre sister, and it was suggested that she should now have an assistant, who, when not needed for duty, could be engaged in the male surgical ward, where extra help was also required. It was also stated that an extra nurse was badly wanted in the women's surgical ward, and ultimately, at the instance of the Chairman, a recommendation that a nurse receiving a salary of £25 a year and an extra probationer be appointed, was unanimously agreed to. The matron was also desired to provide sleeping accommodation outside for the two nurses, there being no room in the Home for them. Under the circumstances this arrangement is, we suppose, unavoidable; but the sooner the authorities of Worcester Infirmary are able to add another storey to the Home, the better. The Chairman says that when the Home was built they never supposed that they were going to do such a vast amount of work; but the tendency with institutions like Worcester Infirmary is, of course, to grow, and when a nurses' home is erected allowance should be made for contingencies during at least a considerable number of years. The practice of sleeping out, even on a small scale and under the best possible conditions, is neither conducive to discipline nor comfort.

### FEMALE TRAIN ATTENDANTS.

For some 18 months past the directors of the Great Western Railway, always solicitous for the comfort of the travellers on their line, have employed on some of their long-distance express trains a female attendant, who is accessible in case of need to all passengers during their journey. One of these quite unusual and useful officials having been described as wearing "a smart nurse's uniform," we have inquired whether they are required to be fully or partially trained nurses. The general manager courteously replies that "such qualification would certainly be in favour of a candidate for the position, but is not essential for the duties which they are required to perform."

### HOLDING INQUIRIES "IN CAMERA."

The proposal of a medical inspector of the Local Government Board for Ireland to hold an inquiry *in camera* into the circumstances under which four nurses recently resigned their positions in the Banbridge Infirmary gave rise to a long controversy. The inspector, in urging that, in the interests of the institution, the investigation should take place with closed doors, suggested that the Guardians should be represented by their solicitor; that the solicitor representing other parties to the inquiry should be present; and also, of course, the persons giving evidence. Individual Guardians, however, protested against what some called "hole-and-corner business," and others a "Star Chamber inquiry"; and it was with some difficulty that the inspector cleared the room and was able to proceed. As a general rule, we are opposed to inquiries *in camera*, especially when the chances are that persons more or less interested will furnish one-sided reports of their own to the press. But in the case of Banbridge Infirmary we can quite understand that a good deal of evidence of public importance, yet calculated to give rise to acrimonious comment, may be given. As proper provision appears to have been made to safeguard the rights of all concerned, we do not think that in this instance any harm will be done by depriving the public of an opportunity of hearing the recital of many more or less personal matters. Of course, a clear statement of the important facts will be published at the termination of the inquiry.

### AN AMALGAMATION OF ASSOCIATIONS.

It has been decided to amalgamate the Bishop Auckland Working Men's Nursing Association with the Bishop Auckland District Nursing Association. This decision is apparently the result of opinions very freely expressed in the town, which, as a matter of fact, is not sufficiently large to maintain two organisations of a similar character. There is no question of the nursing under either being unsatisfactory, and the movement for uniting forces is primarily due to to the failure of the Working Men's Association to obtain enough financial support. With regard to points of difference in methods, these do not appear to us to constitute an adequate reason for maintaining a separate existence, and we hope that the amalgamation scheme will be carried out for the benefit of the working classes generally, and without friction as far as the nurses are concerned.

### PRINCESS LOUISE AND QUEEN'S NURSES IN LIVERPOOL.

The visit of Princess Louise to Liverpool last week was brought to a close by a small function at the Central Home for District Nurses. In response to an enthusiastic greeting, and an eloquent speech from Mr. Archibald Williamson, M.P., Her Royal Highness said that as she herself belonged to the Scottish and also to the Kensington Branch of the Association, she felt quite at home among the members of it in Liverpool. She added that she watched with interest the work in that city, and that the report which had been submitted showed that more had been done than she anticipated. With reference to the report it may be mentioned that last year 8,400 cases were attended and 20,000 visits paid by the nurses, while 57,000 attendances were also registered at the elementary schools. Subsequently, the Princess, though obviously tired after unveiling the statue of Queen Victoria and distributing prizes to the girls at the Liverpool High School, went over the Home, looked at the bed-rooms of the nurses, and also descended to the basement in order to inspect the district room where the clothes for lending the patients and other appliances are kept. The nurses, numbering fifty, were drawn up in the hall and on either side of the wide staircase; they made a pretty and effective picture in their "Queen's" uniform of blue print, with spotless aprons and caps of white. Mr. Williamson presented the matrons of the Association's five Homes, Miss Hills, Miss Wall, Miss Drysdale, Miss Colvin, and Miss Lee, to the Princess, who took tea with the lady superintendent of the Home.

### A FAREWELL FUNCTION AT GUY'S HOSPITAL.

On Thursday last Miss L. M. Tippetts, late assistant matron of Guy's Hospital, started for North India, where she is to be matron of the Government Hospital in Lahore. She was accompanied by Miss Newton, sister of "Lydia" Ward, Miss Morrah, sister of "Charity" Ward, and Nurse Smith, staff nurse, who will also take up work as sisters at the same institution. A farewell service was held on the previous evening in the hospital chapel, which was attended by the matron and most of the sisters and nurses. The nursing and administrative staff presented Miss Tippetts with a dressing-bag as a token of their affection and esteem; the other sisters also received similar gifts from the surgeons and staff of their wards. On leaving the hospital they were heartily cheered by the assembled members of the medical staff; flowers and also a slipper for luck followed in their wake. A party of about twenty proceeded to Paddington Station to complete the send-off.

### MIDWIVES AT ST. PAUL'S CATHEDRAL.

The annual meeting of the Certified Midwives' Total Abstinence League will be held on the afternoon of Friday, October 26, at the Chapter House of St. Paul's Cathedral. The chair will be taken by Dr. Annie McCall, the President, and the speakers will include Miss Le Geyt, Miss Gregory, and Miss May Hughes. All midwives, both members and non-members, also associates, monthly nurses, and any others who care to be present, will be welcomed; and those who would like to visit the crypt should meet Miss Gregory on the steps of the west front of the Cathedral, facing Ludgate Hill, at 2.45 p.m. The meeting will begin at 3.30.

### DEATH OF A MATRON.

The death of Miss Georgina Kinninmont, matron of the West of England Eye Infirmary, Exeter, took place suddenly while she was on her holidays at her home, Levenhall, Musselburgh, and she was buried on Saturday last at Edinburgh. Miss Kinninmont had held her position for ten years, and was exceedingly popular with the patients, as well as with all associated with her. She was instrumental in raising a large amount towards the heavy expenditure entailed in building and furnishing the new wing added to the institution. She will be much missed by the people of Exeter, as well as by the nurses she trained, in whose welfare she always took a kindly interest.

### CLAPHAM MATERNITY HOSPITAL.

We regret to learn that, owing to ill-health, Miss Margaret Patterson has resigned the post of matron of the Clapham Maternity Hospital, which she has held for upwards of five years. Her successor is Miss Bertha Taylor, who has for some time been home sister at the London Temperance Hospital.

### TRYING CASES IN NORTH CORNWALL.

The conditions of district nursing in some parts of North Cornwall still leave a good deal to be desired. Many of the people hold fast to their old notions, some of them not having an idea of how to make a poultice. Diet, they think, is quite a secondary matter, and they have a great dislike to fruit and milk. Their dislike to fresh air is almost as pronounced. We learn, however, from a medical practitioner that the district nurse in Tintagel seems to be doing very useful work in the face of difficulties. This year she has had a number of trying cases. One was that of a woman living in a two-roomed cottage with her son and his wife and seven children, nine sleeping in one room, the patient herself lying on a miserable bed in the living-room. Another was that of a man of 87 in a room about five feet long and five wide; the window had not been open for years. A third was a sufferer from mastitis, almost developed into an abscess, the patient being eventually poisoned by the treatment of a midwife who used dirty rags. These, and the fact that throughout the summer the nurse's day commenced at 7.30 a.m. and ended at 10 p.m., a call out at night not being infrequent, suggest that she does not find much time for recreation or even for rest. We admire the devotion of this nurse, but we wish that she had time for both rest and recreation.

### BEQUEST BY A NURSE.

The late Miss Charlotte Margaret Culverwell, of the private nursing staff of the Bristol General Hospital, who died on September 18, bequeathed to the institution the sum of £50, free of legacy duty. Much gratitude has been expressed by the family of Miss Culverwell to the matron, Miss Morris, and the nursing staff for their kindness during her long and painful illness in the hospital.

### RATEPAYERS AND DISTRICT NURSING.

At a recent meeting of the Swansea Guardians Miss Dillwyn raised an objection to the usual subscription of £75 being paid towards the Swansea Nursing Institute. She maintained that the grant of this money made the whole of the ratepayers contribute towards a charity, whether they wished it or not. By way of rejoinder, it was pointed out that 3,720 visits were paid to parish patients by the district nurses last year, and we think that the Guardians were justified in setting aside Miss Dillwyn's plea.

### SHORT ITEMS.

We are glad to hear that the total amount actually realised by the cricket match organised by Mr. Thomas Hayward in aid of the District Nurses' Institution at Cambridge was £146.—The in-patients of the Cancer Hospital, Fulham Road, were entertained on Monday last by a company of ladies and gentlemen under the direction of Miss Ethel Briscoe.

### The Nursing Outlook.

"From magnanimity, all fears above;
From nobler recompense, above applause,
Which owes to man's short outlook all its charm."

### ADEQUATE AND INADEQUATE TRAINING.

The letter we published last week from "A Matron of Some Years' Experience" on some essentials of sound administration unconsciously reveals the absence of adequate training in the class of institutions to which she refers. Our correspondent's point is that hospitals containing up to 120 beds, if situated in quiet, non-manufacturing towns, do not have sufficient cases to make the training of really efficient sisters possible. In other words the material for training is not forthcoming in these hospitals, and it is not possible, with the restricted material, to fully train probationers in every branch of their profession, or even in the nursing of very many cases which they must have to nurse in private work. Our correspondent declares that the infrequency of acute cases, both medical and surgical, and the absence of the experienced staff nurse who forms so valuable an asset in our large training schools, combine to render it impossible to give probationers at such a hospital a sufficient training to enable them after three years' experience to take even the higher posts, *i.e.* the head nurses' positions in the institutions where they have been nominally trained. Could anything more convincing than this be produced in the way of evidence, to show, that certain institutions which advertise for probationers, and offer to train nurses on terms, have taken up a position which the committees cannot sustain on moral or business grounds? The truth is that the circumstances of such hospitals as the one described by our correspondent, render it undesirable for their committees to attempt to train probationers, because they have neither the material nor the means to give them a complete training. Committees so circumstanced should hesitate to issue a three years' certificate to any probationer whose experience in nursing is limited to that received at any such an institution.

"A Matron of Some Years' Experience" speaks of a county hospital in a non-manufacturing town, but there are a number of county hospitals in the larger towns where every kind of case is admitted to treatment, and where the facilities for thorough training are adequate in all respects. We were more especially referring to these institutions when we dealt with the subjects treated in the article published in our issue of the 22nd ult. There are several county hospitals of the higher type, where promotion is too seldom made to the posts of head-nurse and sister from the staff. We have always maintained that such a system is harmful to the best interests of every hospital, unjust to the nursing staff, and well calculated to irritate and annoy both the honorary medical officers and the nurses, whilst it must tend to diminish efficiency and to interfere with the highest discipline. Our remarks therefore were not only justified but necessary in regard to all hospitals of this type which fail to provide for promotion to the higher posts in the nursing department from the staff trained within the hospital itself.

But what are we to do with these relatively inefficient hospitals and so-called schools for training nurses, to which "A Matron of Some Years' Experience" refers? Our correspondent's letter demolishes their claim to be regarded as nurse-training schools at all under existing circumstances. She claims, no doubt with justice, that the type of women who find their way as probationers to these inadequate establishments are quiet women, anxious to provide for themselves, and capable of making good nurses. For this reason the so-called three years' training, in the conditions named by our correspondent, is accepted by nurses' homes who supply the public with nurses in their own homes, and by the authorities of some workhouse infirmaries, where presumably the incomplete though certificated nurse has an opportunity to complete her training. In every case a nurse who gives her services at a reduced wage to any institution for three years, under an arrangement whereby the institution agrees to train her as a nurse, and to give her a certificate of efficiency at the end of that period, should have a claim against any such institution, if it fails to give her an adequate and complete training in fulfilment of the contract entered into with each probationer at the commencement of the term.

No doubt the present state of affairs has grown up incidentally, and the point may never have been squarely faced or thought out by those immediately responsible. The time has however come when it is essential that every hospital which undertakes to issue a three years' certificate after an equal number of years of adequate training in the duties of a nurse, must be prepared to show that it does provide full and adequate facilities which justify its claim to be considered a training school for certificated nurses. Those institutions which are unable to afford this evidence of competency should co-operate together and group themselves in counties, or by some other means take steps to afford elsewhere to each of their probationers the facilities which they cannot give them in their own etablishment. Failing this they must discontinue to advertise for probationers, to attempt to train nurses and to issue a three years' certificate of training.

### Abdominal Surgery.

By Harold Burrows, M.B., F.R.C.S., Assistant Surgeon to the Seamen's Hospital, Greenwich, and to the Bolingbroke Hospital, Wandsworth Common.

### AFTER-TROUBLES AND COMPLICATIONS OF ABDOMINAL OPERATIONS.

The days following an abdominal operation are never quite free from anxiety, for there is a long list of evil things which may befall the patient. But in most cases these pains and perils may be prevented or ameliorated by intelligent forethought and care.

### Shock.

The preventive treatment of shock has been fully dealt with in a previous article. To lessen its severity the patient's bed should be warmed well before he is replaced in it after the operation, and additional heat should be supplied by hot bottles; but particular care is needed to avoid burning the patient. Unconscious people are apt to receive severe burns from hot bottles inefficiently covered and carelessly applied.

The reason why artificial warmth is so necessary is that a patient in profound shock is practically a cold-blooded animal. Metabolism is nearly arrested, and the tissues are no longer producing the amount of heat which is requisite, so that the temperature of the body falls towards that of the surrounding air.

Next in value to warmth are saline injections. These should be at a temperature of 110° F. when given, and are made by adding a drachm of table salt to a pint of water. They may be injected by the rectum, by subcutaneous infusion, or by introduction directly into a vein. Of these methods the first is the simplest, but it happens not infrequently that injections given in this manner are not retained or absorbed, and so one of the other two methods (preferably the former) must be employed. Subcutaneous injections of saline solution are of the greatest value, and every nurse should know how to administer them.

The materials required are a sterilised douche-can or large glass funnel, two pints of sterile saline solution at a temperature of 115° F., some india-rubber tubing, and a sterilised exploring needle with a large bore. The infusion is usually made under the skin of the chest or abdomen, and, of course, the skin is thoroughly disinfected before the needle is introduced. The needle having been connected with the douche-can by means of the india-rubber tubing, some of the saline solution is allowed to run through until the tube and needle become warm. The needle is then introduced for some distance under the skin at the selected spot, the douche-can is raised about two feet above the level of the needle, and the saline solution allowed to flow. If the apparatus is working properly the skin will be distended by the fluid, so that a swelling appears in the neighbourhood of the needle. If the solution does not run properly it may be that the bore of the needle is too small, or it may have become blocked. In an adult a pint or a pint and a half may be given at a time.

In intravenous infusion the bend of the elbow is disinfected, the vein exposed by a few cuts of the knife, a cannula is introduced into the vein, and the saline solution is allowed to flow in. As the operation will not be performed by the nurse there is no necessity to give a more detailed description of it. But the nurse must see to it that so much of the antiseptic precautions as fall to her must be duly observed, and she must be careful that the saline solution, as it issues from the cannula, is of a proper temperature.

### Vomiting.

Vomiting after an abdominal operation may be due to (1) the anæsthetic, (2) peritonitis, (3) intestinal obstruction, (4) operative accidents, (5) other causes—for example, uræmia.

Vomiting due to the anæsthetic may often be prevented entirely by washing out the stomach before the patient leaves the operating-table. If this be not done there is usually some vomiting. As a rule it is slight and requires no active measures. If it is severe and repeated, gastric lavage should be employed and will almost certainly stop the sickness. A mustard-leaf or hot flannels applied to the epigastrium have been recommended as additional remedies which may be used if gastric lavage is not available.

It is to be remembered that noise and disturbance are factors which tend to increase post-anæsthetic vomiting. For this and for other reasons the patient should be kept as quiet as possible after an operation.

### Tympanites.

By the terms tympanites and meteorism is meant distension of the intestines with gas, the gas being produced by the intestinal bacteria acting upon the bowel contents. To prevent tympanites is one of the main endeavours in the after-treatment of abdominal operations. Occasionally tympanites is severe, the abdomen being so distended that the functions of respiration and circulation are seriously interfered with, the symptoms after a while becoming almost indistinguishable from those of acute, general peritonitis. By that time the intestines will have become paralysed, and any treatment is then too late.

The factors which tend towards the prevention of meteorism are the proper clearing out of the bowels immediately before the operation, the frequent introduction of a rectal tube after the operation, so as to allow flatus to escape, and an occasional alteration of the patient's position in bed. Strychnine is thought to assist peristalsis, and so to overcome a tendency to meteorism, while morphia has the reverse effect. Indeed, it is the belief that morphia favours the production of meteorism, which is the chief objection to its use after abdominal operations.

If the abdomen is becoming distended a timely turpentine enema should be given, followed by the free use of a rectal tube. Unless there has been too long delay, and in the absence of peritonitis, the tympanites is nearly always relieved by this treatment. There is a growing tendency in favour of giving aperients immediately after an abdominal operation, and this practice diminishes the likelihood of severe meteorism.

As a rule, a complaint on the part of a patient that the abdominal bandage is too tight is an indication for the use of a turpentine enema rather than for the loosening of the bandage.

Acute dilatation of the stomach is a rare complication of abdominal surgery. It is manifested by distension of the abdomen and copious vomiting. Treatment frequently repeated consists of gastric lavage by means of a stomach tube.

If a patient begins to vomit after an interval of twenty-four hours or more from the operation, the sickness is of serious import, for it is probably due to peritonitis, intestinal obstruction, or some other complication.

Vomiting after gastro-enterostomy is sometimes benefited by propping the patient up in bed.

### Pain and Discomfort.

Although the degree varies within wide limits, some pain is sure to follow a laparotomy. An easy error is merely to answer a patient's complaint with "I know, there's bound to be pain; but you will very soon feel better," and to let the matter rest with that. Or one may make a rough mental estimation of the patient's pain, and then do nothing or give him morphia, accordingly as the resultant sum is small or large.

But the proper course to take is to consider the possible causes of pain and how they can be removed. Thus pain may be due to a distended bladder, and the catheter will bring a great deal of relief. Abdominal distension is another cause of pain, and, as mentioned above, it can usually be removed by means of a turpentine enema. If there is a drainage tube in the abdominal wound it may be pressing with too much force on some structure, and removal of the pressure is all that is required.

Placing a pillow under his knees will sometimes make a patient more comfortable, and changing his position from time to time may have a similar effect. When being moved on to his side the patient must not be allowed to use any exertion himself, for fear of placing an unnecessary strain on the sutures in the abdominal wound.

### Thirst.

Thirst is a cause of suffering after nearly every case of laparotomy. So long as there is vomiting it is not expedient to meet the complaint by giving the patient water to drink. But to some extent the thirst can be relieved by saline injections given per rectum or subcutaneously. In addition, the patient's mouth and lips should be kept moist. When there is no vomiting, perhaps within five hours from the operation, sips of hot water may be given, cautiously and in drachm doses at first, the quantity being gradually increased as the hours go by if the patient does not vomit; for in most cases sickness is the only contra-indication to the early administration of water by the mouth.

### Sleeplessness and Restlessness.

Pain and discomfort are the chief opponents to the patient's repose, and so the easiest way of dealing with restlessness and sleeplessness is to give morphia. But, as mentioned above, there are objections to this practice as a routine. In any case, before resorting to opiates, it will be well to try other measures and to ascertain if there is any cause which can be readily removed. Further, the restlessness may be consequent upon internal hæmorrhage, and sleeplessness may mark the onset of severe sepsis. These possibilities should pass through the mind and be excluded before opium is allowed to cast its obscuring shadow over the patient.

### Delirium.

It is to be borne in mind that a patient in delirium may jump out of bed and even escape from the ward if the nurse be not watchful. Such a case has happened in my own experience. The patient, a boy operated on shortly before on account of general peritonitis, partly delirious, and suffering from intense thirst, managed to elude the vigilance of the nurse and to escape into the ward bath-room, where he probably hoped to gain the water he so much desired. Little harm was done, but the consequences might have been disastrous. Heart failure or bursting open of the wound might easily be the outcome of such an escapade.

### The Nurses' Clinic.

### THE DISTRICT NURSE AND INFECTIOUS FEVERS.

In large towns the district nurse can rarely undertake to visit the more infectious types of fevers, but in villages it may easily happen that three or four cases of scarlatina, or diphtheria, or an outbreak of measles, may constitute a far greater danger to life and health than all the rest of her patients put together, and therefore the interests of the one class of invalid may without injustice be exclusively considered.

In all circumstances it is indispensable that the district nurse should be able to recognise the early symptoms of infectious fevers, should understand their general course and treatment, their sequelæ, and the precautions that must be taken to prevent the disease from spreading. Recognition of early symptoms is of the first importance; it frequently happens, and not only in very poor homes, that although a child is known to be ailing, no doctor is called in until a fever is well on in its course, and the patient has had every possible opportunity of injuring himself and infecting others. A nurse should keep a keen eye on all the members of the families she visits, especially on children of school age, and never lend an inattentive ear when told of undefined illness in the houses of near neighbours and friends.

Having detected symptoms of infectious fever, the nurse's action with regard to undertaking the case must, of course, depend upon her situation, but she should never leave the house without seriously warning the parents of the dangers to all concerned, and advising them as to isolation, feeding, and general treatment. If she has reason to believe that they are neglectful, and that no doctor will be sent for, she should at once communicate with the medical officer of health. If the family circumstances are bad and isolation practically impossible, the nurse should do all in her power

### THE NURSES' CLINIC—*Continued.*

to persuade the parents to send the child to a fever hospital, assuring them that the charge will be strictly proportioned to their means, that the patient will be attended by specially skilled persons, and will have an infinitely better chance not merely of life, but—which is of more lasting importance—of complete recovery. In the case of very young, timid, and much petted children, the risk of "fretting" must not be lost sight of, although, as a rule, it is much smaller than tender mothers like to believe, and usually more than counterbalanced by the readier obedience given to strangers in the matter of food, medicine, etc.

If the parents are determined to keep the child at home, and are legally entitled to do so, the nurse should first turn her attention to the choice and arrangement of the sick-room. The largest and airiest upstair room should be chosen. Considering the crowded state of most poor men's dwellings, it is not always practicable to clear the patient's room completely of all needless articles of furniture, but nothing must be retained that cannot be easily and completely disinfected.

Although a low temperature is needed for the earlier stages of most fevers, a fire in the patient's room is almost indispensable. In all illness there is constant need of hot water, clean linen, etc., and if there is no fire at hand there will be such an incessant coming and going that isolation is reduced to a mere farce. The window can, of course, be well opened, provided there is no draught, the fire needs only be very small, and the heat, if distressing to the patient, can easily be screened off. A sheet dipped in disinfectant and constantly kept wet should always hang over the door.

The room must be kept thoroughly clean and free from dust, and the floor and all woodwork should be washed over with disinfectant every day. Dusters should be damped with disinfectant, and afterwards soaked in a strong solution. All bed and body linen used by the patient must either be soaked in disinfectant or immediately boiled with disinfectant soap. Handkerchiefs are a frequent source of infection owing to the carelessness with which they are treated. Evacuations of every kind need specially strong solutions of disinfectant; the doctor usually states its nature and strength, but in most cases we have to rely on whatever is supplied freely by Town Hall or Vestry.

Special cups, tumblers, plates, etc., must be kept for the patient (I have often found half the family crockery in the sick-room), and washed in his room with a special basin and cloth. If the patient is very sensitive to noise, the basin can be lined with a cloth folded in four, and only one article should be washed at a time. After washing, china, glass, and plate should be kept completely immersed in cold water until required.

In very poor houses it is often practically impossible to avoid keeping food in the patient's room, but it can at least be well covered up and placed where he cannot be annoyed or disgusted by constantly seeing it. In warm weather it is a good plan to put two or three inches of cold water in a clean bath, stand the food in it in basins, and cover the bath with a cloth wrung out in cold water. The food most commonly ordered for fever patients, milk, beef tea, broth, eggs, etc., are all liable to rapid deterioration in a warm temperature, and great care must be exercised to insure that nothing in a doubtfully wholesome state is given to the patient. A definite place must be fixed on for medicines, food, and everything used for the invalid; this will save a considerable amount of work and make fussiness and disorder impossible. The patient's friends must be warned never to eat or drink in the sick-room, and to be scrupulously careful as to the condition of their hands.

In most cases of fever the light, whether natural or artificial, needs to be subdued, but it should always be possible for the patient to know whether it is night or day; sleep, as in most forms of illness, is of more consequence than food; or, to put it more correctly, it is possible to supply food at any hour of night or day, and therefore postponement of meals is often of little importance, but sleep cannot be supplied to the patient in this easy fashion, and he must therefore be allowed to rest undisturbed. The nurse must, of course, be able to distinguish between genuine sleep and a motionless, apathetic state caused by extreme weakness. In the latter case food must be given with unbroken regularity. If the patient is unable to take sufficient food, or strongly opposed to doing so, unusually large amounts of alcohol may be needed, but on this point the doctor must be consulted. It must be remembered that differences of constitution maintain themselves in all circumstances, and some persons endure a high temperature and very little food for a considerable period without being in peril of their lives, while others are quickly reduced to a dangerous state of exhaustion.

If delirium is likely to occur, the relations should always be warned, and if they are unaccustomed to illness, its nature and the right way of treating it should be carefully explained. Only a few months ago I found a most high-principled and religious old man regarded with the utmost horror owing to the mutterings of delirium. Even his wife, who had lived with him for over forty years, told me plainly, "I thought it was just his wickedness!" Unless the ravings are of a wildly irrational type, I have known their character to be entirely mistaken, even by experienced nurses.

When a case of fever is considered to have terminated, the nurse must speak seriously (and in some houses repeatedly) of the sequelæ of the disease, the aftermath of evil results that may so very possibly be heavier than the original crop, and detail the precautions needed, whether abstention from study, over exertion, or indigestible food, avoidance of chills, or whatever it may be. If the poor could but once be persuaded that one cannot with regard to fevers, "have 'em and ha' done with it," a great step in advance would have been taken.

### Incidents in a Nurse's Life.

### A MOTHER.

It was nearly midnight. All the lights were out in the big ward except the shaded one on the table; but the moonlight streaming in through the windows and lying in white patches on the polished floor, gave an unearthly radiance and lit up the worn faces of the sleeping women with a weird beauty. The night-nurse might have been a ghost, she moved about so silently. She was glad that the patients were all asleep; there was time to watch the moonlight and the drifting clouds. The street below had not yet settled down to the short silence of a London night; she could hear the voices of men and women quarrelling, shrieks of hideous laughter, and now and then a little child's voice crying. She looked down and saw a woman standing in the middle of the road. She had evidently been fighting, for her clothes were torn and her hair hung in wisps round her flushed, angry face. Two children clung sobbing to her skirts. "A mother is a mother still, the holiest thing alive." The line came involuntarily into the night-nurse's mind as she turned away from the window, and she wondered if Coleridge had ever seen a mother like that.

"Nurse!" called a little sharp voice, and the night-nurse ceased wondering and went across to the first bed in the ward. A little girl lay there, or, rather, sat quite upright, supported by a perpendicular pile of pillows. Her head was thrown back, showing the painful pulsation and the distended veins in her neck. Her little soft child's face was discoloured by a much disorganised circulation, but it was a dear little face still, and the scattered hair on the pillow was soft and golden. Her breath came in short gasps, making every word, every movement an exertion to be considered. "Has Mammie come?" she asked, and there was a thrill of intense anxiety in her voice that hurt the night-nurse.

"Not yet, Annie," she said.

"Then she won't now," said Annie. "I knew she wouldn't."

"Oh! perhaps she will—she said she would," said the night-nurse, forgetting the street outside and going back to her own recollections of a mother.

Annie looked up scornfully at the night-nurse; she was very skilful at arranging pillows and making the strenuous work of respiration as easy as possible, but how little she knew of the world after all! It was Saturday night; Annie knew where her mother was, and why she had not kept her promise and returned. Little Annie! she was only eight, and she supposed it was the right place for mothers to go to on Saturday night—what she would probably do when she was a mother; but that did not make it less sad for the little children who were ill and wanted them.

She closed her eyes and two tears rolled down her cheeks. "I knew she wouldn't come," she said again.

The night-nurse was very fond of Annie, and she could not bear to see the tears. She wished she could be her mother for one night.

"Don't cry, Annie," she said, bending over her; "don't cry, darling! Try to go to sleep. She will be sure to come in the morning"—and, with a sudden inspiration,—"You can have some bacon for breakfast if you like!"

Annie opened her eyes again; they were very wide and blue, and the moonlight shone right into them—she was like a picture of an angel.

"And a fried egg as well?"

The question was hardly in keeping with the angel eyes, but the night-nurse was very pleased to hear it. She smiled down at her and answered, "Yes."

Annie was comforted. She put her head back and closed her eyes, for she was very sleepy.

The night-nurse kissed her forehead and said softly, "Good-night, my sweet!"

The blue eyes looked up once more with a wonderfully radiant smile.

"Good-night," said the quick, panting voice. She wanted her mother, but she liked the night-nurse and she recognised that she had tried to do her best.

The next night Annie's mother sat beside her. She was a stout woman, dressed in greasy black clothes, and the atmosphere around her was by no means fresh. Her face was red and hot-looking, and she was continually dabbing it with a dirty, black-edged pocket-handkerchief. Her eyes were blood-shot and full of tears, though it was difficult to tell whether they were the result of emotion or of the preceding night's entertainment. The night-nurse, who liked to think the best of people, inclined to the former theory.

Annie made no conjectures. She accepted her mother with placid content. The tears, the long-drawn sighs, the dirty handkerchief, and the greasy clothes were all familiar to her, much more familiar than was the gentleness of the night-nurse or the tender voice that had said, "Good-night, my sweet!" There was the strange strong tie of kinship between this woman, surely one of the lowest of human beings, and the little fair dying child, who was possessed of so much faithful affection and resolute courage.

Annie was much worse, and very drowsy; but she kept rousing at intervals and opening her eyes to see if her mother was still there. Experience in the past had made her distrustful, and the vigilance with which she watched her would have been amusing if it had not been so sad.

All through the long night scarcely a word passed between them. Frequently Annie wanted a drink or to be lifted up higher; but each time the night-nurse had to do it, for she vigorously refused to let her mother touch her. Yet each time she turned to her afterwards with her quick radiant smile, as though to explain that it was not because she did not love her best, but only because she recognised the great need of trained skill and dexterity. And each time the black-edged handkerchief came greatly into use, and the night-nurse had to move away quickly.

The last time came just before five, when the sparrows were chirping on the window-sills and the fresh Spring dawn was breaking. The ward was very silent, even those who had had but a wakeful night—for they were all fond of Annie—had fallen asleep.

"Lift me up 'igher," panted the distressed little voice.

The night-nurse hesitated and looked across at the poor mother, surely now——

"No! you!" came imperatively. The night-nurse raised her in her arms; her face was ghastly, but she made one more effort. She put her little hand into her mother's and turned her head feebly towards her with that wonderful smile of sweet apology.

A few minutes afterwards a sound of loud sobbing came from behind the screen, the sunlight streamed into the wards, the sparrows chirped more loudly on the window-sills, and the other patients awoke. But Annie's short and painful life was over.

### Examination Questions for Nurses.

### OPENING OF THE AUTUMN SESSION.

Once more we begin our Examination season. This will be the ninth year that we have occupied the respective positions of candidates and examiner. I hope that holidays have proved beneficial to all, and that now all nurses are returning refreshed and benefited to take up once more their interesting but arduous work. With regard to the examinations, there is a distinct improvement in the quality of the answers sent in; but in this world nothing stands still, either we go forward or we slip back—let the former condition of things be ours.

The chief faults still observable are first, a want of applying practical common sense to the matter in hand; secondly an inclination sometimes on the part of the writer to leave her own province of nursing pure and simple and to enter that of the medical man; and, thirdly, a tendency to discursiveness—a wandering on to branch lines of wordiness and sometimes even of sentimental twaddle, instead of keeping strictly to the business under discussion. Let me once more warn all intending competitors to read the rules carefully and to take pains as to the tidiness and legibility of their papers; a nurse who writes, folds, and posts her work in a slovenly manner is likely also to be slovenly in all things. What is worth doing at all is worth doing well, and she who is faithful in little will also be faithful in much.

Nurses will be perhaps surprised to hear that every year I receive from between twenty and thirty papers without name or address!

Question for October.

In a case of inguinal or femoral hernia (in private or district work) what steps should you take to alleviate suffering pending the arrival of the doctor?

Rules.

The competition is open to all. Answers must not exceed 500 words, and must be written on one side of the paper only, without divisions, head lines, or marginal notes. The pseudonym, as well as the proper name and address, must be written on the *same* paper, and *not on a separate sheet*. Papers may be sent in for 15 days only from the day of the publication of the question. All illustrations strictly prohibited. Failure to comply with these rules will disqualify the candidate for competition. Prizes will be awarded for the best two answers. Papers to be sent to "The Editor," with "Examination" written on the left-hand corner of the envelope.

In addition to two prizes honourable mention cards will be awarded to those who have sent in exceptionally good papers.

N.B.—The decision of the Examiner is final, and no correspondence on the subject can be entertained.

Any competitor having gained three prizes within the current year shall be disqualified from taking another until 12 months shall have expired since the first prize was gained.

### Quarantine at the Antipodes.

It was our first experience, and we thought it grand. A patient had contracted plague, and we were isolated from that time. We were not even allowed to write our letters, which was our only drawback. The sister gave us permission for suppers, dances (fancy dress and plain), novelty teas, and lawn card-parties at all hours after duty. How we did enjoy ourselves! But how queer to see no fresh faces admitted into our wards, no ambulances driving up our drives, and no visitors. Rules and regulations after duty were for once thrown aide; with sister's permission, doctors, surgeons, dispensers, and nurses were all as friendly as if such a thing as strict hospital etiquette had never been known. We were even allowed up till 10.30 p.m. instead of 10. Our evenings on the lawn were lovely; it being full moon, small groups of nurses with their particular friends brought out their cards and enjoyed a game by the light of the moon. After the nights began to get dark we used to fossic out bicycle lamps, etc., to light up the lawn; and with the aid of a few Japanese lanterns the effect was very pretty. We always finished up the "crib" or "euchre" parties with supper, and were never short of cakes, as we could telephone for them; and I am afraid that our bills were very large ones at the cake shops that month. Whilst our respective parents were eagerly scanning the daily papers for some news regarding the outbreak (to say nothing of worrying themselves about us), we were thoroughly enjoying ourselves and having a real royal time. No honoraries to worry us, no new cases coming in, no operations, and only a few patients! Even the troopers on patrol outside our gates were envious, and one was heard to say that he wished he could have been put on duty inside the hospital instead of out. Occasionally we put through the iron railings to those strict officers of the law some supper, which they, after a hurried glance around, would avail themselves of, and enjoy it, too. At the expiration of a few weeks of dissipation our gates were once more open to the public; but no visitors availed themselves of the privilege for several days. They seemed frightened at the very sight of us. It was laughable, when we were allowed to go out in the street, to do some shopping. We were not permitted to visit our friends for some time after. We were amazed to see folks hurry by and give us a very wide berth; when they saw a uniform approaching it seemed as though we had the street to ourselves.

It took us all a little time to settle down again to the usual routine and discipline, and for months afterwards we were always on the look-out for more quarantine.

### German Midwives in Conference.

The Annual Conference of the German Association of Midwives took place at the end of last month at Dusseldorf. The Federation includes 228 societies and comprises 11,301 members, more than 400 of whom took part in the Conference.

With the object of bringing about uniform regulations for midwives throughout the entire empire it was decided to hold the conference in future in a different State every year.

The district physician, Dr. Krohne, of Dusseldorf, who also represented the Dusseldorf authorities of the Congress, spoke on the new regulations for midwives in Prussia, and stated that the Ministry was at present engaged in the preparation of a law on the subject which would have the effect of improving the economic position of midwives.

In the course of the discussion the idea was repeatedly expressed that midwives should receive a suitable compensation for the expenses entailed upon them through the new regulations, which should be paid by the State which had issued them.

This proposal was embodied in a resolution, "That a petition should be drawn up claiming for midwives the position of public officials paid and pensioned by the State." This was carried unanimously.

The question of the danger to the health of nurses through poisonous disinfectants, especially sublimate, was thoroughly discussed. A demand was also raised that when the sanitary authorities required the disinfection of the residence of a midwife and of her kit, the cost of the same should be borne by the local authorities and not by the midwife.

Professor Schlossmann, of Dusseldorf, spoke on natural and artificial feeding of infants. The main idea of his address was that it was the duty of midwives to see not only that the child was born alive and without injury, but also that it remained alive and developed into a strong and healthy human being, who would eventually be of service to the State. For that object the best means which could be employed was the natural breast-milk of the mother. Midwives, therefore, should have it imposed upon them as a duty to do everything in their power to persuade mothers to feed their children themselves. Midwives and nurses should work for this object by imparting all the necessary instruction and explanation. It was a mistake to prescribe for the nursing mother what she should eat. Nursing mothers should eat anything which agreed with their taste and their digestions. The speaker finally urged that at the lying-in hospitals the pupils, in addition to their training in midwifery, should pass through a probationary course in the care of infants.

There was also a discussion as to the advisability of instituting courses of post-graduate lectures for midwives, among those who spoke being Dr. Frank, the Director of the Midwifery School for the Province of Cologne.

### Presentations.

Charing Cross Hospital.—Miss Esther Hodges, ward sister at Charing Cross Hospital, has been presented with a silver tea service, a carriage clock, and beaten bronze tea-caddy by the matron, sisters, and nurses, on the occasion of her appointment to the post of matron of the Buchanan Hospital, St. Leonards. Miss Hodges carries with her the good wishes of all with whom she has worked during the eight years she was at Charing Cross Hospital.

### Practical Hints.

We welcome notes on practical points from nurses.

### SOME USES FOR BRAN.

This cheap and useful article might be more used with great advantage both in private and hospital nursing. Bran-pads of various sizes and thicknesses are a great comfort to patients. Two made oblong, say fourteen by six inches, not too thick, placed under the buttocks relieves the pressure from the backbone, or are useful when reading if placed under the elbow. Smaller ones can be utilised to rest sore heels, a depression, of course, being made in the centre. In confinements one fair-sized one put under the binder is most comfortable, being firm yet yielding. If there is much pain the bran-pad may be made thoroughly hot between plates in an oven, or, if the plates are enamel, over a gas-ring or spirit-lamp, then wrapped in flannel and placed on the abdomen. A lightly-made one was found very efficacious in a case of "appendicitis." These pads remain hot a long time. Small ones are also a boon when tiny babies have stomach ache. It is a pity that bran-pads are not adopted in hospitals instead of rings made of more expensive wool, tow, and bandage, which so soon become hard and flat. If a variety of bags were kept made amongst the ward stores ready to be filled with bran as required they would be found most efficacious and economical. They are quickly made and are very light. The writer has found these pads of great use with her own patients. To those in pain even the smell of the hot bran is soothing and comforting. In private houses I usually make use of white flannelette or soft long-cloth, which should always be washed. It has the advantage that when soiled the bran can be emptied out and the bag washed.

### Everybody's Opinion.

[Correspondence on all subjects is invited, but we cannot in any way be responsible for the opinions expressed by our correspondents. No communication can be entertained if the name and address of the correspondent are not given as a guarantee of good faith, but not necessarily for publication. All correspondents should write on one side of the paper only.]

### THE SITUATION AT THE NURSES' HOSTEL.

Miss C. Brittlebank, Nurses' Hostel, writes: Will you allow me to express through your columns the great kindness and courtesy I have received for many years, both at Percy Street and the Nurses' Hostel, from Miss Wood and Miss Paul, and the regret I feel at their resignations? When asked by a nurse to sign a petition, I refused for the simple reason that I had too much confidence in and respect for Miss Wood, and I can never sufficiently show my gratitude to her for having founded the Nurses' Hostel and for the many privileges I have enjoyed when staying there.

"L. S." writes: Reading the account in last issue of Miss Hulme's dismissal, it may interest others, and perhaps help to console her, that someone else is being quite as unjustly discharged. I have been staying in a convalescent home near Bath, where the matron did her best to make everyone comfortable and feel at home. She received by post notice to leave, as the committee found that she had not carried on the Home in accordance with their rules. I believe that, with one exception, the committee go to the Home once a year, leaving the hon. secretary to manage or mismanage the Home. All that he will tell the matron is that letters of complaint have been received. If these were put before the committee, why not letters of thanks and gratitude? Surely, before being dismissed by a committee, an individual should be given some chance of justifying herself.

"A Nurse" writes: On reading your article about the situation at the Nurses' Hostel, Francis Street, one is struck by the extraordinary apparent injustice to Miss Hulme, who has been asked to resign at a week's notice for no adequate reason. I say "no adequate reason" as surely Miss Wood's statements about "lack of discipline" and a "disposition to stir up strife," both of which can be easily refuted by Miss Hulme, cannot be called adequate causes for a week's notice? It is odd, surely, to talk of "lack of discipline" in an hotel except amongst the employés, but this seems to refer to the nurse visitors at the hostel; and as to "stirring up strife," anyone who knows Miss Hulme in the least knows that her presence always seems to quell strife, and that she would never countenance resistance to authority, and she was, during the period she held office, most loyal to Miss Wood's wishes. Always kind and courteous to all, she was not a person who could be imposed upon.

"An Employer of Nurses" writes:—As a shareholder in the "Nurses' Hostel" and superintendent of a private nursing institution, I must express my indignation at the manner in which Miss Hulme has been treated. The matters brought to her notice in the petition presented by the nurses have long been an abuse at the hostel, so much so that I, though a shareholder, have advised my nurses not to live there, and I had made up my mind that if I could attend the annual meeting I should lay these matters before the members. If our money is to be wasted in this high-handed manner and no public explanation given as to the reason for Miss Hulme's dismissal, with three months' salary in advance, the sooner we sell our shares the better. I have myself asked on the telephone for nurses and been told that they were out, afterwards finding that they had been in all the time, and as even I am not allowed to speak to nurses on the telephone I have to bring them a long way to receive their orders before going to cases; moreover, at night the telephone is taken off at 11 p.m., so that if a nurse is wanted after that hour a messenger has to be sent, thus wasting valuable time in cases which are always urgent at night.

"J. M. T." writes from the Nurses' Hostel:—I wish to make a few remarks on a matter which is the subject of much comment just now, and would be glad if you would kindly give them a place in your journal. I allude to the changes which have taken place recently in the Nurses' Hostel. From the articles, letters, etc., which appeared in various publications last week, it would seem that the nurses were unanimous in criticising and demanding an explanation of Miss Wood's line of conduct. The real truth is that a large proportion remained loyal to her and her colleagues, and had perfect confidence that, whatever course she thought it right to take, she had good and sufficient reason for. I may say that these were, without exception, nurses who had known Miss Wood longest, and had had most dealings with her, whereas the malcontents numbered a large proportion of new-comers. Miss Wood, in her courtesy, acceded to their desire, and gave as full and clear an explanation of the whole proceeding as any one who had brains to understand could desire, while it left their esteem and respect for the late lady superintendent quite unshaken. I cannot conclude without testifying personally, in the most heartfelt way, to the countless kindnesses I have received from Miss Wood and her staff, and to their ready compliance with any reasonable request.

"B." writes:—May I inquire what is meant by Miss Hulme being dismissed for "want of discipline" with the nurses at the Hostel, Francis Street? Are nurses at this hostel different to any other people? Nurses pay for all they receive. If one nurse shares a room with another they each pay 17s. 6d. per week. This does not include boots, bath, or late leave. If she visits a theatre or is out one hour after 11 o'clock, which is closing time, she has to pay 1s., while if a whole party are out 6d. a head is charged.

May I ask your readers if they have ever heard of a manageress being dismissed from an hotel for being courteous, for that is what Miss Wood seems to mean by want of discipline. Miss Hulme was courteous and kind to every nurse in the place. Would it not be well for the new lady superintendent to spend six months in one of H.M. prisons before taking up her duties, as discipline appears to be the only thing needed at this hostel? She might then be known as the "mother of correction" instead of the lady superintendent. We are told that the question of Miss Hulme's dismissal was considered by the Board for two hours. Surely some of the members will give us further information. May I, for instance, ask if Miss Hulme was allowed to appear at this important meeting to justify or defend herself? It is strange that the nurses at the Hostel saw no signs of the disposition to stir up strife of which Miss Wood speaks, and stranger still that with these two traits in her character that she should have won the love and respect of the nurses.

"A Nurse in the North" writes :—My sympathy for Miss Hulme has been aroused by the treatment she has received at the hands of her "board." I have no personal interest in Miss Hulme, and am a perfect stranger to her, but I well remember my first and only attempt to make use of the hostel. I arrived there at 6.30 a.m., having travelled all night; I had engaged a room some three months before and also written a few days before to give notice of my coming. Upon arriving I was told that my name was not down for a room, but it might be at the New Block. I inquired there, and was told the same thing. I went back to the hostel and, after waiting half an hour, was told that Miss Wood (to whom I had written) and Miss Paul would not be in until 8 o'clock. I waited at the New Block until after 8 o'clock, and was told to go down to breakfast. By the time I had persuaded the maid to find me a bedroom in which to leave my luggage it was 8.30, and on going down to the breakfast-room found it locked, and was told that it was against the rules to give breakfast after meal hours. I was caused much inconvenience by the letters not being put out punctually, and was altogether most uncomfortable. To crown this I was charged almost double what I ought to have been (this was made good afterwards on the remonstrance of the friend who advised me to go to the hostel). After my experience the first morning I took no meals in the hostel except breakfast. I thought then it was quite time that some change was made in the management, particularly as nurses are shareholders, and I would urge the nurses to press for an inquiry into the entire conditions of the working of the hostel, which ought to be, but is not, a "home" for nurses.

"A. M. J." writes :—As one who has stayed at the Nurses' Hostel, I wish to add my protest against the unjust and incomprehensible dismissal of Miss Hulme. I can only account for it by supposing that the directors wish to keep this institute under the insufferable conditions of the past, for it is the general feeling of the nurses who have been in residence since Miss Hulme was appointed lady superintendent that they have never before been treated with such tact and kindliness. Even in her short time with us several improvements in the management of the hostel have occurred. That these were necessary I will point out by one brief example of the absurdity of some of the unwritten rules which successfully prevent any of us feeling a sense of home in this place. The first time I entered the hostel, cold from a long journey, I found that I was just in time for afternoon tea, and was thankful to follow my companions into the dining-room at once. Scarcely had I seated myself at one of the tables than a maid-servant approached and ordered me to "Take off my hat." This, I was informed, was one of the rules! I need scarcely add that a rule so ridiculous could hardly be printed! My room, I found, was at the top of the house, but my box was at the bottom, and, it appeared, that it was one of the rules that no nurse should have her box in her own room! These petty and tyrannical regulations, of which there are many, have succeeded in making the hostel uncomfortable—scarcely better than a girls' school. But we are grown and reasonable women, who ought not to be subjected to such indignities.

### INSPECTORS OF MIDWIVES.

"A North-West Midwife" writes : Comparing notes with a friend of mine the other day respecting the rules of the Central Midwives Board and the trials and tribulations of midwives in trying to live up to the letter of the law—no easy task nowadays—I recalled my first fear of an inspector. I pictured something of the male species, supercilious and overbearing. Instead, a lady called. When I say a lady I mean a lady in every sense of the word. On her card was "Miss P——, Inspector of Midwives," and instead of the much-dreaded supervising authority whom I had expected, a lady with a firm, nice face and kindly brown eyes came towards me, speaking in a business-like manner, with a pleasant reassuring voice. Miss P—— carefully looked up my case-book, made some inquiries, found fault with some of my arrangements, looked at my bag and appliances, suggested some improvements in the arrangement of things, made note of other items, then quietly said "Good morning," and left me with an impression that the midwives under Miss P——'s supervision are to be congratulated. If they do their duty they are sure to have the advice and help of a trained, cultured, and experienced woman, and one who, while insisting on the rules of the Central Board being rigidly carried out, will do it with a firm graciousness—a good way of enforcing the respect and gaining the confidence of the practising midwives under her supervision.

### TENDER FEET.

"S. F." writes : In a recent issue there appeared an article on the "Care of the Feet," and I now venture to suggest a trial of a simple and efficacious remedy for tender feet. This recipe is particularly noteworthy of nurses and those who have long hours of standing. Wash the feet with soap and warm water in the ordinary way, but do not soak them. Immediately after drying with a towel pour into the palm of the hand a little neatsfoot oil, and well rub over each foot, and also round the ankles. Then put on clean socks or stockings. The ease and comfort derived from this treatment is surprising, the benefit lasting some days. Neatsfoot oil can be obtained at most druggists, and in small quantities, which will last some time, very little being required when used. The oil also has a good effect on corns and hard skin on the feet.

### SECTARIANISM IN LONDON HOSPITALS.

"Alpha" writes : In reply to "Medicus" *re* Sectarianism, there are, I believe, not a few nurses who have been made to suffer because of their adherence to the Catholic faith. I know of several, and I also speak from experience. I was trained in a large London training school where it was the rule—when there were vacancies—to promote the senior staff nurse to the post of sister. But when, as happened more than once, the "senior" happened to be a Catholic, the post was kept open till she had resigned, and then given to the next. Looking back, I remember well how I hoped on day after day that I would be offered the vacant sister's post. I had been very happy during my training, and I felt that my efforts to do my best had been appreciated, as I had been given excellent ward and theatre work and sister's duties; and my ambition was to be the first Catholic sister in that institution. But I, too, was passed over, and I felt it very much. I know that in many of the hospitals Catholic probationers are taken, and in some they are exempt from the compulsory attendance at prayers and church services; but whether they are often favoured with promotions or not I cannot say.

### THE PAY OF NURSES.

"Observer" writes : An article appeared in your paper a few weeks ago on the subject of nurses' pay. Why is it so small? The average amount is generally £35 inclusive, and yet nurses work at a high pressure—often 11½ hours daily, and sometimes longer (including Sundays), with the strain of night duty frequently added. What would the bank clerk, with his usual eight hours a day, and the majority of working men, with their week-ends at home, say to above conditions? I fancy that there would soon be a change in affairs. Why, many men can make as much money in a few months as a hard-working nurse can hope to do in the same number of years. The fact is that many nurses are too anxious to appear Christian martyrs. Surely they can act it, and yet be ambitious enough to have what is their due, for the sake of the future. Nursing has been changed greatly from what it used to be, but there is still room for a few improvements. "Trained nurses" come under a higher standard than the cook and untrained nurse-attendant, and yet these are frequently offered the same salary. I maintain that even if a nurse be very moderate in her ways, it will take her all her time to save a very little out of such a salary for a rainy day with a few comforts. And that is supposing she is in possession of good health and joined to some institution "above board." In private work there are nursing homes good, and nursing homes bad. How is a well-trained nurse to choose one from the other? Unfortunately, she often makes a mistake, which I can prove, because I have met those nurses. Why is there not a system like the General Post Office, with London head-quarters and branches for private nurses, so that really efficient, trained nurses can take their proper place instead of joining nursing homes, which are often "shady"? If the Government would only take this up it would be a mercy to many nurses and the public at large, who would be free from the "untrained humbug" at last.

### Appointments.

[No charge is made for announcements under this head, and we are always glad to receive and publish appointments. The information, to insure accuracy, should be sent from the nurses themselves, and we cannot undertake to correct official announcements which may happen to be inaccurate. It is essential that in all cases the school of training should be given.]

ACLAND NURSING HOME, OXFORD.—Miss Effie White has been appointed assistant matron. She was trained at the Bristol General Hospital. She has been attached to a private nursing home in London, and has since been sister of the medical wards of the Royal Hospital for Sick Children and Women, Bristol.

CLAPHAM MATERNITY HOSPITAL.—Miss Bertha Taylor has been appointed matron. She was trained at the London Temperance Hospital, and has since been sister in various wards of this institution, finally occupying that of home sister.

DUDLEY POOR-LAW INFIRMARY.—Miss Ada Strivens has been appointed charge nurse. She was trained at the Rochdale Union Infirmary, and has since done private nursing. She has also temporarily taken head nurse's duties at Tadcaster Poor-law Infirmary.

STANLEY HOSPITAL, LIVERPOOL.—Miss Mary Fraser has been appointed sister. She was trained at Wolverhampton General Hospital, and has since been night sister at the Guest Hospital, Dudley.

STOCKPORT INFIRMARY.—Miss J. G. Stronach has been appointed sister. She was trained at Stanley Hospital, Liverpool, and has since been staff nurse and theatre sister in the same institution.

YEOVIL DISTRICT HOSPITAL.—Miss Eva Lucy Goring has been appointed staff nurse. She was trained at Lowestoft Hospital, and has since been staff nurse at Carshalton and District Hospital, the Northwich Victoria Hospital, and sister at Surbiton Hospital.

### Novelties for Nurses.

(BY OUR SHOPPING CORRESPONDENT.)

### CIRCASSIAN UNDERWEAR.

MESSRS. GREENSMITH, DOWNES AND SON, of 143 George Street, Edinburgh, have a wonderfully large and excellent selection of warm materials for underwear. Their newest production is Circassian pure wool underwear. This is made of an elastic material which is manufactured in a pale grey and cream mixture. Special pains have been taken in the matter of shape, the armholes are ventilated, and it is guaranteed not to shrink. The price is moderate. Especially fascinating are the mixtures in silk and wool. Amongst them is a cellular material. The Australlama is a fine combination of cashmere wool and Australian merino. The price-list, entitled "A book of underwear," affords a revelation of what is possible in materials for underclothing.

### To Nurses.

WE invite contributions from any of our readers, and shall be glad to pay for "Notes on News from the Nursing World," "Incidents in a Nurse's Life," or for articles describing nursing experiences at home or abroad dealing with any nursing question from an original point of view, according to length. The minimum payment is 5s. Contributions on topical subjects are specially welcome. Notices of appointments, letters, entertainments, presentations, and deaths are not paid for, but we are always glad to receive them. All rejected manuscripts are returned in due course, and all payments for manuscripts used are made as early as possible after the beginning of each quarter.

### TRAVEL NOTES AND QUERIES.

BY OUR TRAVEL CORRESPONDENT.

THE BLACK FOREST (Inquirer).—I think, on the whole, Hornberg would be the best place, because you are on the actual Schwarzwald line, which enables you to make numerous inexpensive excursions. Where all is beautiful it is difficult to say in a hard and fast manner which is the best spot, but with your requirements I fancy Hornberg would suit you. The money is divided into marks not francs, and the rate is a little higher. A mark is, roughly speaking, the same as a shilling. At Hornberg you would be received (at the season you mention) on terms beginning at 4 marks, either at the Hôtel Bär or Hôtel Post. Triberg, with its renowned waterfall, is very near, but it is a dearer place to stay in than Hornberg. If you like to divide your time between two different places St. Blasiew is cheap; apply to the Pension Waldeck. Or at Todtmoos (a lovely spot) apply to Villa Bellevue, or Pension Schmid. Your second question is a little obscure. Is it Pensions in Venice you want? If so, I know of nothing quite so cheap as you need. Try the Pension Lewald, 743 Fondamento S. Vio, or Pension Bril-Daru, Tragetto S. Gregorio, Grand Canal.

RULES IN REGARD TO CORRESPONDENCE FOR THIS SECTION.—All questioners must use a pseudonym for publication, but the communication must also bear the writer's own name and address as well, which will be regarded as confidential. All such communications to be addressed "Travel Correspondent, 28 Southampton Street, Strand." No charge will be made for inserting and answering questions in the inquiry column, and all will be answered in rotation as space permits. If an answer by letter is required, a stamped and addressed envelope must be enclosed, together with 2s. 6d., which fee will be devoted to the objects of "The Hospital" Convalescent Fund. Ten days must be allowed before an answer can be published.

### Notes and Queries.

**REGULATIONS.**

**The Editor is always willing to answer in this column, without any fee, all reasonable questions, as soon as possible.**

**But the following rules must be carefully observed.**

1. **Every communication must be accompanied by the name and address of the writer.**
2. **The question must always bear upon nursing, directly or indirectly.**

**If an answer is required by letter a fee of half-a-crown must be enclosed with the note containing the inquiry.**

*Nursing Abroad.*

(1) Are there any hospitals in Paris or Gibarltar where English nurses are employed?—*Madrid.*

The Hertford Hospital, Paris. We cannot help you as to Gibraltar.

*Monthly Nursing.*

(2) I was trained as a monthly nurse at a nursing home in 1893. I am now anxious to put my knowledge into practice, but I am told I must pass an examination before I shall be allowed to practise. What am I to do, and to whom should I apply?—*Hampstead.*

You may still practise as a monthly nurse. Probably your informant was talking of midwifery, which may not be practised without the certificate of the Central Midwives Board. We strongly advise you to join an institution to brush up your knowledge, for it is not likely a medical man would recommend you without recent experience.

*Paralysis.*

(3) Can any of your readers suggest how to keep a paralysed lady dry who lies continually on her side and suffers from incontinence?—*Matron.*

The constant use of sanitary towels, frequently removed, and the parts dusted carefully with some preparation, like Mennen's toilet powder; undivided attention to the draw-sheet; the skin under all circumstances must be kept dry. The case is eminently one requiring a skilful and attentive nurse.

*Corns, Collodion, and Biniodide.*

(4) (1) I have heard that sea water is good for feet with corns. Can you tell me whether the boxes of prepared sea salt would answer the same purpose? (2) That collodion painted on tender feet is a great relief. Is there any danger to the inflamed foot by the use of collodion if the skin is not broken? (3) Is there any form of biniodide in which instruments can be placed without rusting?—*Inquirer.*

(1) Water dressing is the best treatment for corns. Soaking the feet night and morning in warm water, with or without the addition of sodium carbonate. Some medical men advise 15 minutes' soaking twice a day; the principle is the same, *i.e.*, the addition of alkaline salts to the water, the effect of which is seen on the hands of bottle-washers. Also see letter to-day in our Correspondence Column on "Tender Feet." (2) Collodion is not recommended. (3) Do not place instruments in biniodide.

*Rome.*

(5) Is there a home employing English nurses in Rome?—*Bird of Passage.*

The Anglo-American Nursing Home, 265 Via Nomentana.

*Guy's Hospital.*

(6) After training at a provincial hospital is a nurse allowed to enter Guy's hospital for a year—not as a probationer—and then belong to the Institute for their nurses?—*M. B. B.*

Nurses who wish to join the Institute enter into an agreement for four and a half years—three years' training at the hospital and one and a half year to work for the institution.

*Cancer.*

(7) Is there a cancer hospital where a poor woman of seventy suffering from cancer can be received free?

The Cancer Hospital, Fulham Road, Brompton, S.W.; but patients must provide clothing and pay for laundry. Write to the Secretary.

**Handbooks for Nurses.**

| | Post Free. |
|---|---|
| "A Handbook for Nurses." (Dr. J. K. Watson) ... | 5s. 4d. |
| "Nurses' Pronouncing Dictionary of Medical Terms" | 2s. 0d. |
| "Art of Massage." (Creighton Hale) ... ... ... | 6s. 0d. |
| "Surgical Bandaging and Dressings." (Johnson Smith.) ... ... ... ... ... ... | 2s. 0d. |
| "Hints on Tropical Fevers." (Sister Pollard.) ... | 1s. 8d. |

Of all booksellers or of The Scientific Press, Limited, 28 & 29 Southampton Street, Strand, London, W.C.

### For Reading to the Sick.

"THE COMPANY OF ANGELS."

O Archangel of Compassion!
Unto thee God's Heart is given;
For thou lov'st the gifts of healing,
Most of all the gifts of Heaven.
O thou human-hearted Seraph!
How I long to see thy face,
Where in silver showers of beauty
God bedews thee with His grace!
But I see thee now in spirit
'Mid the Godhead's silent springs,
With a soft eternal sunset
Sleeping ever on thy wings.

*F. W. Faber.*

The prince of guardian spirits, the guardian angel of all humanity, is Raphael; and in this character, according to the early Christians, he appeared to the shepherds by night "with good tidings of great joy, which shall be for all people." It is, however, from the beautiful Hebrew romance of Tobit that his attributes are gathered: he is the protector of the young and innocent, and he watches over the pilgrim and the wayfarer. The character imputed to him in the Jewish traditions has been retained and amplified by Milton: . . . his sympathy with the human race, his benignity, his eloquence, his mild and social converse.—*Mrs. Jameson.*

And we know not how far the ministry of angels may be employed in higher transactions. We know not what part they may bear, under the presiding agency of God the Holy Ghost, in the conveyance of spiritual blessings to the soul. The grace which rouses and strengthens the will, the mercy which lifts us up after our falls, and is felt as an additional incentive to prayer and holy effort, the peace which simple faith in Christ brings into the soul, and which results also from bearing lovingly His light yoke and easy burden—we are unable to say, because we are so ignorant of the nature of spiritual transactions.

The foot of the great bright ladder, whose top reaches to heaven, is in our very midst, stretching upwards with its shining stair to the Throne of God; and around it, and upon it, are the blessed angels, waiting, to carry up our prayers to God, and to fetch down to us the Divine benediction. Will you charge them with no message in behalf of yourself? Shall they not have the joy of seeing you throw yourself at the feet of the present Saviour, and abandon yourself to the treatment of the good Physician?—*Dean Goulburn.*

The Divine existence multiplies itself. The company of spiritual beings who surround Him with their loyalty and love, the angels in countless orders sweeping upward from the ministers of man's lower wants up to those who stand nearest the throne—all these in some belief or other have been included in the faith of every race of men, of almost every man, who had come to the knowledge of a spiritual world and trusted in a God. We must not rob ourselves of the strength and richness that the thought of their existence has to give.—*Bishop Phillips Brooks.*

And have not we sore need the faith to hold
Of the surrounding of the angel bands;
'Mid all earth's dust to trace their steps of gold,
And feel the uplifting hands?—
To feel them near in hours of toil and weeping;
With reverence hail each soul's celestial guest;
Till they shall come, the final Harvest reaping,
To fold us into rest.

*E. Brine.*

